# The Impact of Immunomodulatory Treatment on Kappa Free Light Chains as Biomarker in Neuroinflammation

**DOI:** 10.3390/cells9040842

**Published:** 2020-03-31

**Authors:** Franz Felix Konen, Ulrich Wurster, Torsten Witte, Konstantin Fritz Jendretzky, Stefan Gingele, Hayrettin Tumani, Kurt-Wolfram Sühs, Martin Stangel, Philipp Schwenkenbecher, Thomas Skripuletz

**Affiliations:** 1 Department of Neurology, Hannover Medical School, 30625 Hannover, Germany; konen.felix@mh-hannover.de (F.F.K.); wurster.ulrich@mh-hannover.de (U.W.); jendretzky.konstantin@mh-hannover.de (K.F.J.); gingele.stefan@mh-hannover.de (S.G.); suehs.kurt-wolfram@mh-hannover.de (K.-W.S.); stangel.martin@mh-hannover.de (M.S.); schwenkenbecher.philipp@mh-hannover.de (P.S.); 2 Department of Clinical Immunology & Rheumatology, Hanover Medical School, 30625 Hanover, Germany; witte.torsten@mh-hannover.de; 3 Department of Neurology, University of Ulm, 89081 Ulm, Germany; hayrettin.tumani@uni-ulm.de

**Keywords:** Kappa free light chains, multiple sclerosis, pre-analytic impact factors, biomarker, intrathecal synthesis, cerebrospinal fluid, serum

## Abstract

Background: Kappa free light chains (KFLC) are a promising new biomarker to detect neuroinflammation. Still, the impact of pre-analytical effects on KFLC concentrations was not investigated. Methods: KFLC concentrations were measured in serum and cerebrospinal fluid (CSF) of patients with a newly diagnosed multiple sclerosis (MS) or clinically isolated syndrome (CIS) before (n = 42) or after therapy with high-dose methylprednisolone (n = 65). In prospective experiments, KFLC concentrations were analyzed in the same patients in serum before and after treatment with high-dose methylprednisolone (n = 16), plasma exchange (n = 12), immunoadsorption (n = 10), or intravenous immunoglobulins (n = 10). In addition, the influence of storage time, sample method, and contamination of CSF with blood were investigated. Results: Patients diagnosed with MS/CIS and treated with methylprednisolone showed significantly lower KFLC concentrations in serum as untreated patients. Repeated longitudinal investigations revealed that serum KFLC concentrations continuously decreased after each application of methylprednisolone. In contrast, other immune therapies and further pre-analytical conditions did not influence KFLC concentrations. Conclusion: Our results show prominent effects of steroids on KFLC concentrations. In contrast, various other pre-analytical conditions did not influence KFLC concentrations, indicating the stability of this biomarker.

## 1. Introduction

An immunoglobulin synthesis within the central nervous system is frequently observed in a broad spectrum of autoimmune and infectious neurological diseases [1,2]. When multiple sclerosis (MS) is suspected in patients with a single clinical episode, cerebrospinal fluid (CSF) investigation usually follows magnetic resonance imaging [1]. According to the latest 2017 revision of the McDonald criteria for MS, the detection of an intrathecal immunoglobulin production as measured by oligoclonal bands restricted to CSF can substitute as a criterion to demonstrate disseminated inflammation in time and thus establish the diagnosis [3]. Oligoclonal bands can also serve as a biomarker to stratify the risk for patients after a single clinical episode to develop MS [4,5]. Although the determination of oligoclonal bands is currently the gold standard to detect intrathecal immunoglobulin G production, the method of isoelectric focusing with consecutive silver staining or immunoblotting is time and cost consuming and requires experiences in the interpretation of the results [6,7]. In search of alternative biomarkers, the determination of free light chains was in the focus of several studies [8,9]. The two isotypes kappa and lambda light chains are components of immunoglobulin molecules and are also secreted by plasma cells as free light chains alongside the production of intact immunoglobulins [10,11,12]. However, although being a promising biomarker for MS and other neuroinflammatory diseases, kappa free light chains (KFLC) are currently not established for clinical routine due to missing methodological standards [13]. Since immunomodulatory treatment with intravenous corticosteroids, immunoadsorption, plasma exchange, and intravenous immunoglobulins is often started before taking CSF samples in patients with severe neurological disability, the knowledge of the effects of such treatments on the concentration of KFLC is of great importance but scarcely examined. We thus investigated in detail if such therapies might influence the reliability of KFLC as a biomarker. We further assessed if storage time, sample method, and contamination of CSF with blood should be taken into consideration when determining KFLC.

## 2. Methods

### 2.1. Retrospectively Collected Data

Medical records were screened for patients who presented with symptoms suggestive for a first demyelinating episode at the Department of Neurology of the Hannover Medical School between 2010 and 2015. Patients were included when they were either newly diagnosed with MS according to the 2017 McDonald criteria or converted to MS during follow-up. Some of these patients were previously investigated with a focus on different other aspects [14,15,16,17,18]. A total of 107 patients were included. Paired CSF and serum samples that were collected as part of clinical routine were utilized. Methylprednisolone was given at a dose of 1000 mg per day for 3 days in one group of patients and 5 days in another group of patients without oral tapering. None of the patients were treated with intravenous methylprednisolone before nor were any of the patients on oral corticosteroid therapy. Most patients received a lumbar puncture before methylprednisolone treatment. In the other patients, serum and CSF samples were collected after 1000 mg, 2000 mg, 3000 mg, 4000 mg, or 5000 mg of a high-dose intravenous methylprednisolone therapy. The retrospective part of this study was approved by the institutional ethics committee of the Hannover Medical School (No. 7837_BO_K_2018, 6 April 2018).

### 2.2. Prospectively Collected Data

In the prospective part of the study, serum samples were taken from patients who were either treated with intravenous methylprednisolone, immunoadsorption, plasma exchange, or intravenous immunoglobulins at the Department of Neurology of the Hannover Medical School in the time from 2018 to 2019. For the analysis of pre-analytic variables, CSF and corresponding serum samples originated from patients that underwent routine lumbar puncture between 2018 and 2019. All prospectively investigated patients gave their informed consent for inclusion before they participated in the study. Demographic data of these patients are depicted in Table 1 and Supplemental Table S1.

#### 2.2.1. Intravenous Methylprednisolone Treatment

In the group of patients treated with intravenous methylprednisolone, all 16 patients received 1000 mg methylprednisolone per day for at least 3 days, while 9 of these patients were treated for an additional 2 days. Serum samples were taken before treatment as well as after 24 h and 48 h from all 16 patients. In addition, blood was sampled after 96 h from 9 patients who were treated five days with steroids.

#### 2.2.2. Plasma Exchange Therapy

Plasma exchange therapy was administered for at least 3 cycles with 1 cycle each day for all 12 patients, while 7 of these patients received 2 additional cycles. Serum samples were taken before treatment, after 24 h, 48 h, and 72 h from all patients and additionally from 7 patients after 96 h and 120 h after the start of plasma exchange.

#### 2.2.3. Immunoadsorption Therapy

All 10 patients who were treated with immunoadsorption received 5 cycles of therapy in total with 1 cycle each day. Serum samples were taken before and after 24 h, 48 h, 72 h, 96 h, and 120 h of treatment.

#### 2.2.4. Intravenous Immunoglobulin Treatment

Serum samples were taken from 10 patients who were treated with intravenous immunoglobulins. Samples were taken before the treatment with immunoglobulins was started and within 12 h after the last infusion of the cycle with cumulative dose of 60–160 g immunoglobulins.

#### 2.2.5. Pre-Analytic Conditions

This part of the study aimed to investigate whether contamination with 5000 erythrocytes per mL CSF or 20,000 erythrocytes per mL CSF might cause an influence on KFLC concentrations (n = 17). Further, CSF and serum samples were stored for 14 days at 4 °C and room temperature and subsequently compared with baseline values (n = 16). EDTA and serum samples of the same patients were compared to investigate the influence of different sample methods on KFLC concentrations (n = 33).

### 2.3. Analytical Procedures

For the retrospectively collected data, all paired CSF and serum samples were analyzed according to routine diagnostic procedures in the Neurochemistry Laboratory of the Department of Neurology. CSF cells were counted manually using a Fuchs–Rosenthal counting chamber. Total protein in CSF was determined by Bradford dye-binding procedure in centrifuged samples [19]. Concentrations of albumin, immunoglobulin type G (IgG), IgM, and IgA in serum and CSF samples were measured by kinetic nephelometry (Beckman Coulter IMMAGE, Brea, CA, USA). Intrathecal synthesis of IgG, IgA, and IgM was calculated according to Reiber’s revised hyperbolic function [20]. Isoelectric focusing in polyacrylamide gels with consecutive silver staining was used to detect CSF-specific oligoclonal bands [21]. KFLC concentrations in CSF and serum samples were determined by nephelometry with N Latex FLC kappa Kit (Siemens Healthcare Diagnostics Products GmbH, Erlangen, Germany) according to the manufacturer’s instruction on a BN Prospec analyzer (Siemens Healthcare Diagnostics Products GmbH). Since the concentration of KFLC in CSF depends not only on the intrathecal produced fraction but also on diffusion of KFLC from blood across the blood–CSF barrier, the correction formula (KFLC CSF/KFLC serum)/(Albumin CSF/Albumin serum) was used that takes the albumin quotient as a marker of the blood–CSF barrier function into account. The calculated KFLC index was considered as elevated above the empirically defined threshold >5.9 [8]. Analyses performed at the Neurochemistry Laboratory of the Department of Neurology were regularly evaluated as a quality control measure by the external INSTAND survey program for analytic methods [22].

### 2.4. Statistical Analysis

GraphPad Prism version 5.02 was used for statistical analysis. The level of statistical significance was set to 5%. Data were described by medians, standard deviation, and interquartile ranges. The D’Agostino and Pearson omnibus normality test was used to asses for normal distribution of values. Mann–Whitney U-Test was used for independent values. Kruskal–Wallis test and Friedman test with Dunn’s Multiple Comparison post hoc test were used for group comparison and Wilcoxon signed-rank test and paired t-test for comparison of two groups with repeated measurements.

## 3. Results

### 3.1. Mean Serum KFLC Concentrations are Lower in Patients Treated with Methylprednisolone 

**Figure 1:**
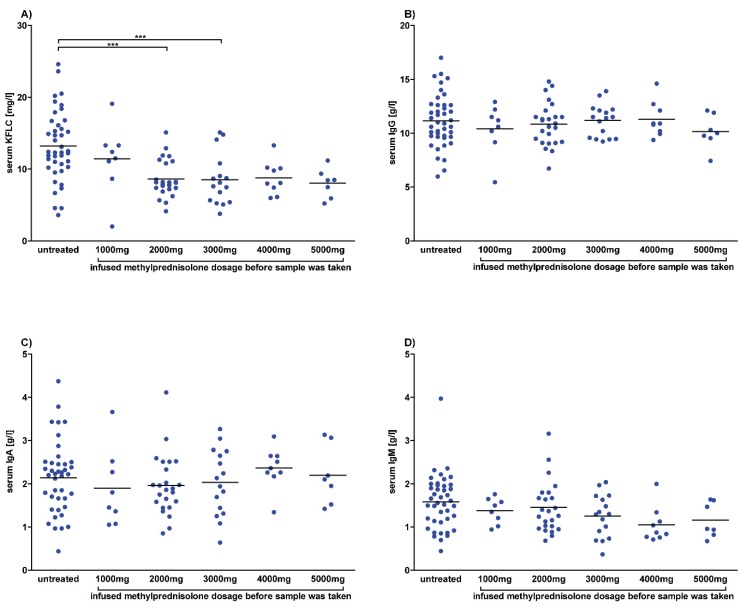
Serum concentrations after methylprednisolone. Serum concentrations of patients diagnosed with multiple sclerosis according to the McDonald criteria of 2017 and clinically isolated syndrome who converted to multiple sclerosis during follow-up. Each dot represents a single measurement of a patient who was untreated or received intravenous methylprednisolone with the indicated dose. Depicted are serum concentrations of kappa free light chains (KFLC) (**A**), immunoglobulin type G (IgG) (**B**), IgA (**C**), and IgM (**D**) in untreated patients (n = 42) and patients who received methylprednisolone infusion of 1000 mg (n = 8), 2000 mg (n = 25), 3000 mg (n = 16), 4000 mg (n = 9), or 5000 mg (n = 7). *p*-values of significant differences are illustrated as asterisks.

**Figure 2:**
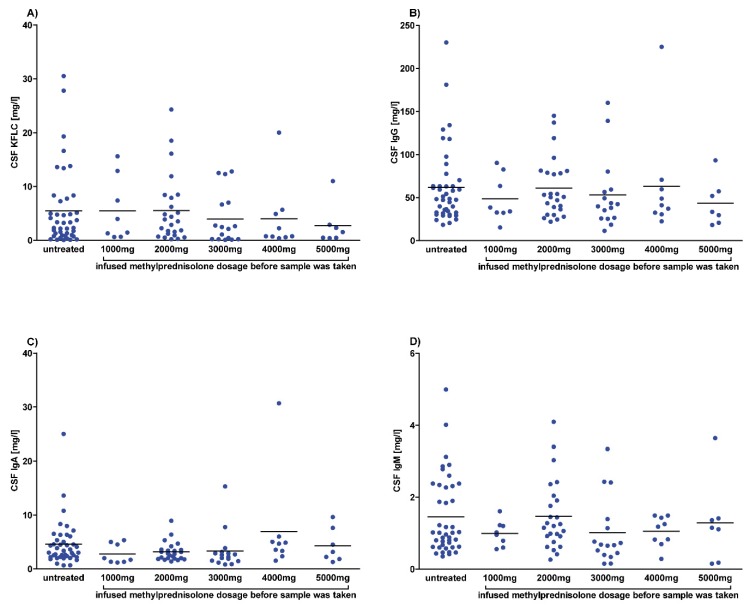
Cerebrospinal fluid (CSF) concentrations after methylprednisolone. CSF concentrations of patients diagnosed with multiple sclerosis according to the McDonald criteria of 2017 and clinically isolated syndrome who converted to multiple sclerosis during follow-up. Each dot represents a single measurement of a patient who was untreated or received intravenous methylprednisolone with the indicated dose. Depicted are CSF concentrations of kappa free light chains (KFLC) (**A**), immunoglobulin type G (IgG) (**B**), IgA (**C**), and IgM (**D**) in untreated patients (n = 42) and patients who received methylprednisolone infusion of 1000 mg (n = 8), 2000 mg (n = 25), 3000 mg (n = 16), 4000 mg (n = 9), or 5000 mg (n = 7). *p*-values of significant differences are illustrated as asterisks.

The results of retrospectively analyzed serum and CSF are shown in Figure 1 and Figure 2 and Supplemental Figure S1. The mean serum KFLC concentration of untreated patients was higher than in patients who received methylprednisolone and reached significant differences between untreated patients and patients who received a total dosage of 2000 mg (*p* < 0.0001) and 3000 mg (*p* = 0.0009) methylprednisolone. In contrast, the mean serum concentrations of the immunoglobulins IgG, IgA, and IgM and of albumin did not differ significantly between untreated patients and patients who received methylprednisolone.

In CSF, the mean KFLC concentration was slightly lower in patients after 3000 mg, 4000 mg, and 5000 mg methylprednisolone but this did not reach significant differences. In addition, no differences were found between mean CSF concentrations of the immunoglobulins IgG, IgA, and IgM and of albumin between untreated patients and patients who received methylprednisolone.

Calculating the KFLC index revealed slightly higher mean values for patients who were treated with methylprednisolone than for untreated patients but did not reach significant differences. The KFLC index was above the threshold of 5.9 in 60/65 patients who were treated with methylprednisolone and in 36/42 patients who were untreated. No significant difference between the quotients of IgG, IgA, and IgM was found between treated and untreated patients (Supplemental Figure S2).

### 3.2. Serum KFLC Concentrations Continuously Decrease with Increasing Steroid Dosage 

**Figure 3:**
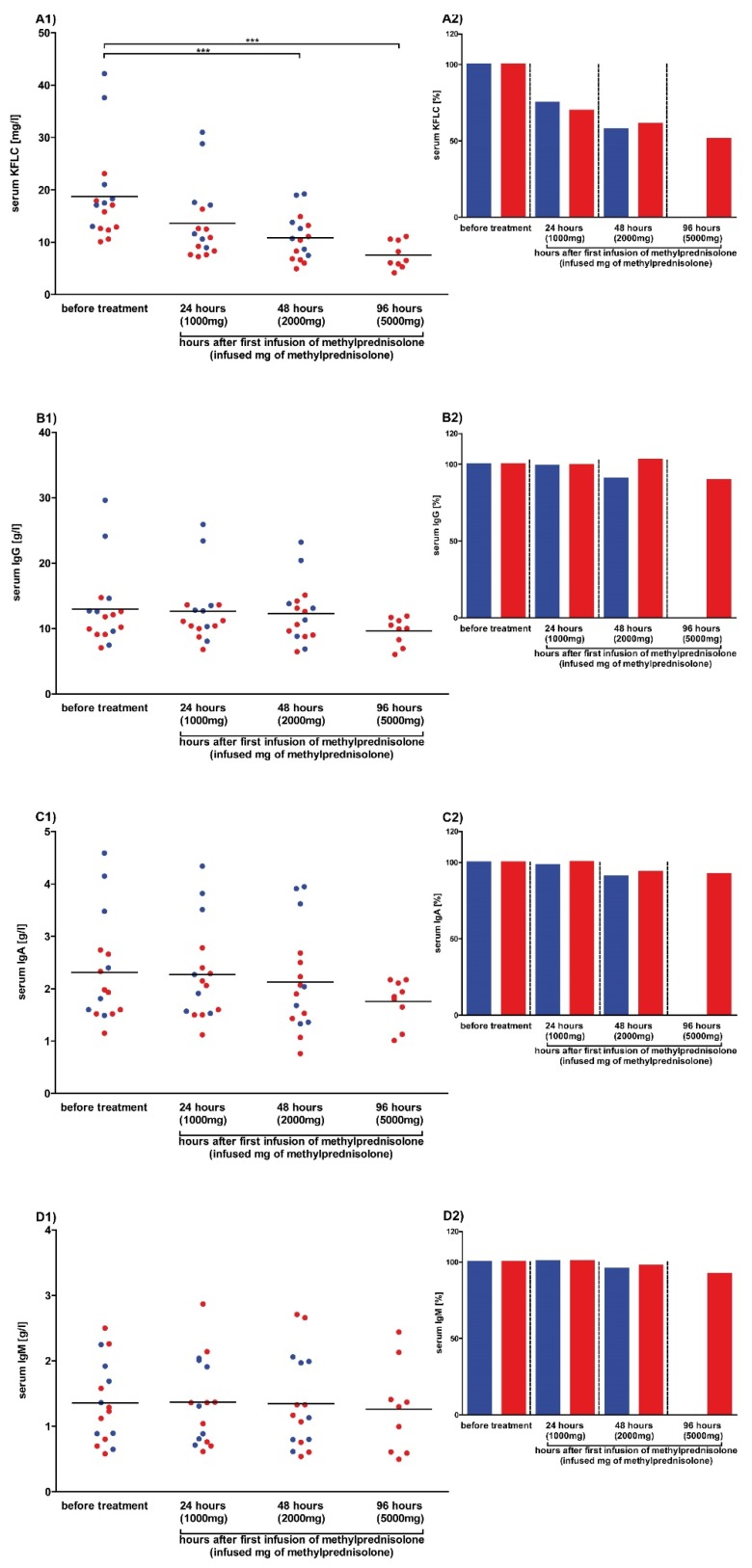
Serum concentrations after methylprednisolone in prospective patients. Serum concentrations of patients whose data and samples were collected in 2018 and 2019 are shown. Depicted are serum concentrations of kappa free light chains (KFLC) (**A1**), immunoglobulin type G (IgG) (**B1**), IgA (**C1**), and IgM (**D1**). The percentage decrease of concentrations of KFLC (**A2**), IgG (**B2**), IgA (**C2**), and IgM (**D2**) in serum is also shown. Patients treated with intravenous methylprednisolone for 3 days are depicted as blue dots and columns (n = 7), while patients with a treatment of 5 days are depicted as red dots and columns (n = 9). Blood samples were taken before and after 24, 48, and 96 h of treatment with 1000 mg of intravenous methylprednisolone per day. *p*-values of significant differences are illustrated as asterisks.

To analyze the effects of high-dose steroids on serum KFLC concentrations, serum samples were collected before therapy and after 24 h and 48 h in each patient (n = 16) treated three days with 1000 mg methylprednisolone per day. Furthermore, serum was sampled after 96 h from 9/16 patients who were treated five days with steroids. Our results show that serum KFLC concentrations continuously decreased after the increasing dosage applications of methylprednisolone. Significant differences were found between baseline KFLC values (18.7 ± 9.1 mg/L) and KFLC values measured after 48 h (10.9 ± 4.4 mg/L) and 96 h (7.6 ± 2.6 mg/L). The mean percentage decrease of the KFLC concentration in serum after 24 h was 28% (± 9%), after 48 h 40% (± 12%), and after 96 h 49% (± 10%) of the initial concentration, independently whether a high or a low concentration was measured before treatment. While the mean serum concentrations of the immunoglobulins IgG, IgA, and IgM and albumin decreased slightly after each application of methylprednisolone, the differences reached significant levels for albumin after 96 h only as depicted in Figure 3 and Supplemental Figure S3.

### 3.3. Plasmapheresis Has No Impact on Serum KFLC Concentrations

**Figure 4:**
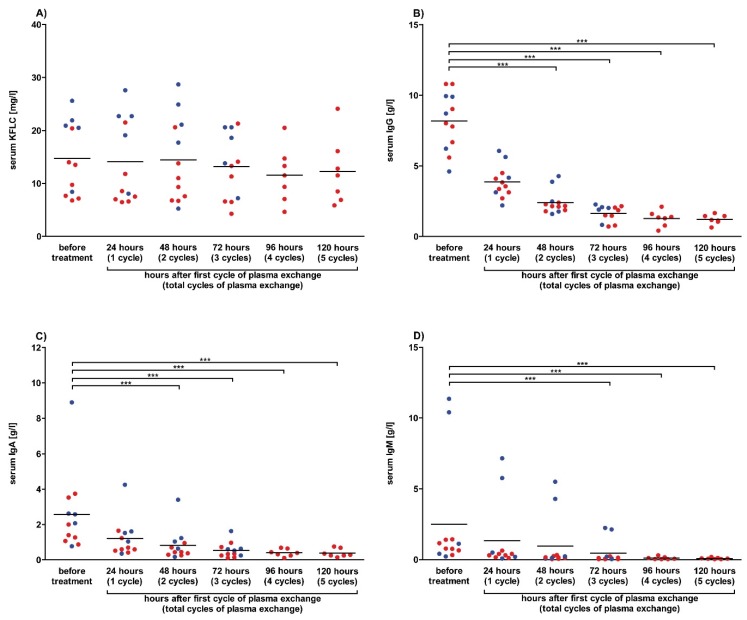
Serum concentrations after plasma exchange in prospective patients. Serum concentrations of patients whose data and samples were collected in 2018 and 2019 are shown. Depicted are serum concentrations of kappa free light chains (KFLC) (**A**), immunoglobulin type G (IgG) (**B**), IgA (**C**), and IgM (**D**). Patients treated with 3 cycles of plasma exchange therapy are depicted as blue dots (n = 5), while patients with 5 cycles of treatment are depicted as red dots (n = 7). Blood samples were taken before and after 24, 48, 72, 96, and 120 h of treatment with one cycle of plasma exchange per day. *p*-values of significant differences are illustrated as asterisks.

Serum samples were collected before therapy and after 24 h, 48 h, and 72 h in each patient (n = 5) treated three times with one plasma exchange therapy per day in order to assess the impact of plasma exchange on serum KFLC concentrations. Furthermore, serum was sampled after 96 h and 120 h from 7/12 patients who were treated with five cycles of therapy. Our results show no significant effects of plasma exchange therapy on serum KFCL concentrations. In contrast, the concentrations of the immunoglobulins IgG, IgA, and IgM decreased after each cycle of plasmapheresis and reached significant levels (Figure 4). High concentrations of KFLC were found in patients with paraproteinemia, which is in line with excessive amounts of immunoglobulins (IgA or IgM) in three patients (Figure 4C,D). The concentration of serum albumin increased significantly after plasma exchange, which might be explained by substitution of albumin after each therapy cycle (Supplemental Figure S3).

### 3.4. Immunoadsorption Has No Impact on Serum KFLC Concentrations

**Figure 5:**
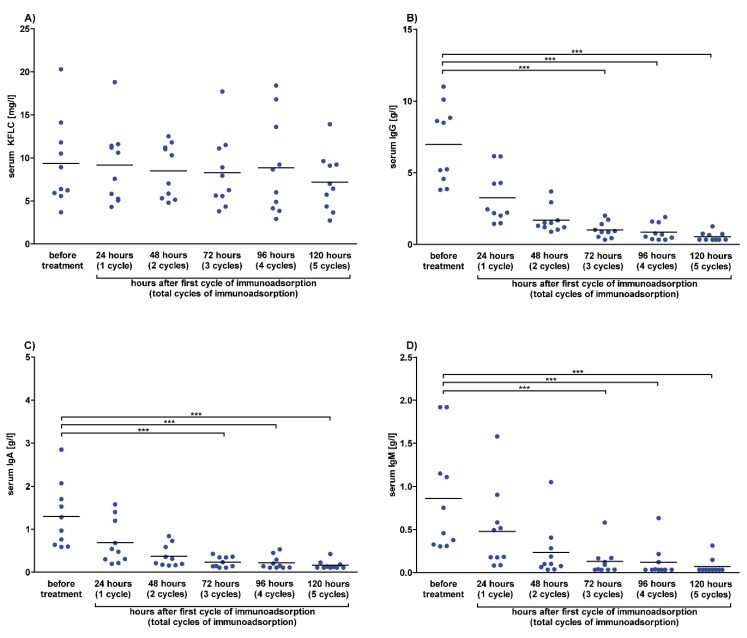
Serum concentrations after immunoadsorption in prospective patients. Serum concentrations of patients whose data and samples were collected in 2018 and 2019 are shown. Depicted are serum concentrations of kappa free light chains (KFLC) (**A**), immunoglobulin type G (IgG) (**B**), IgA (**C**), and IgM (**D**). Blood samples were taken before and after 24, 48, 72, 96, and 120 h of treatment with one cycle of immunoadsorption per day (n = 10). *p*-values of significant differences are illustrated as asterisks.

The influence of immunoadsorption on serum KFLC concentrations was investigated in serum samples that were collected before therapy and after 24 h, 48 h, 72 h, 96 h, and 120 h in each patient (n = 10) treated five times with one immunoadsorption therapy per day. The effects of immunoadsorption on serum KFLC and immunoglobulin concentrations were comparable to the effects of plasmapheresis. Serum KFLC concentration decreased slightly after each cycle of immunoadsorption but did not reach significant differences. The concentrations of the immunoglobulins IgG, IgA, and IgM decreased after each cycle of plasmaphereses and reached significant differences (Figure 5). Concentrations of serum albumin decreased as well and were significantly lower after 72–120 h of immunoadsorption (Supplemental Figure S3).

### 3.5. No Effects of Intravenous Immunoglobulins on Serum KFLC Values

**Figure 6:**
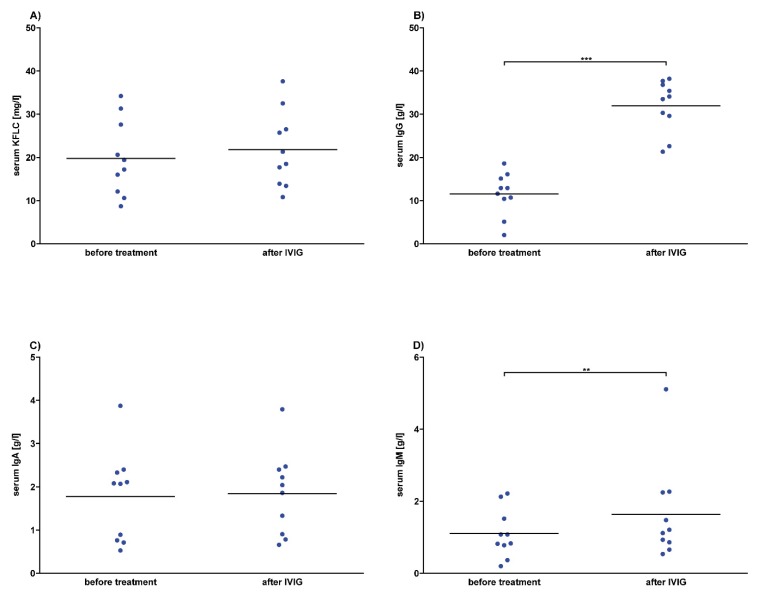
Serum concentrations after intravenous immunoglobulin (IVIG) in prospective patients. Serum concentrations of patients whose data and samples were collected in 2018 and 2019 are shown. Depicted are serum concentrations of kappa free light chains (KFLC) (**A**), immunoglobulin type G (IgG) (**B**), IgA (**C**), and IgM (**D**). Blood samples were taken before and after treatment with intravenous immunoglobulin in a dosage between 30 g and 40 g per day, resulting in a total dosage of 60 g–160 g (n = 10). *p*-values of significant differences are illustrated as asterisks.

To analyze the effects of intravenous immunoglobulins (IVIG) on serum KFLC values, serum samples were collected before and after two to five days of a daily infusion therapy of 30 g to 40 g of intravenous immunoglobulin in 10 patients. Our results show no significant effects of intravenous immunoglobulins on KFLC concentrations; however, a tendency to higher KFLC concentrations in serum after the infusion was observed (Figure 6). Since intravenous IgG immunoglobulins were infused, the concentration of serum IgG increased significantly. The serum concentration of IgM increased as well, while IgA values remained stable and serum albumin decreased significantly (Figure 6 and Supplemental Figure S3).

### 3.6. KFLC Are Stable Despite Various Pre-Analytical Effects

Different pre-analytical conditions were investigated as a possible influence on serum and CSF KFLC concentrations. Measurements of serum and EDTA samples from the same patients did not differ in KFLC concentrations (Supplemental Figure S4A). Storage of serum samples at 4 °C and room temperature for 14 days resulted in similar results indicating the stability of KFLC for at least two weeks (Supplemental Figure S4B). Similar to blood, CSF KFLC values were not influenced when stored at 4 °C or room temperature (Supplemental Figure S4C). Furthermore, addition of blood to CSF (containing 5000 and 20,000 erythrocytes per mL CSF) did not change results of KFLC measurements (Supplemental Figure S4D).

## 4. Discussion

The main finding of this study is that intravenous steroids significantly influence the concentrations of KFLC in serum. KFLC concentrations continuously decreased as a consequence of increasing dosage applications of methylprednisolone. The amount of KFLC decreased to 58% after 48 h and further to 41% after 96 h as compared to values before therapy with steroids. The previously unknown influence of steroids on the concentration of KFLC is an important issue since high-dose methylprednisolone is the treatment of choice in various neuro-immunological disorders especially in events suggesting MS [23,24].

The presence of oligoclonal bands in CSF, which indicate an intrathecal immunoglobulin synthesis, is a hallmark biomarker of MS and has been demonstrated to be highly prevalent [17,25,26]. Consequently, since the 2017 McDonald criteria, oligoclonal bands can be used as a criterion to demonstrate disseminated inflammation in time to diagnose MS [3]. Beside intact immunoglobulins, plasma cells secrete free light chains as well, which might be an additional biomarker [8,11]. However, the impact of pre-analytical influences such as relapse therapies on KFLC concentrations has not been investigated to date.

In clinical practice, therapy with methylprednisolone often starts in patients who present with symptoms indicating MS such as optic neuritis even before the complete diagnostic work-up including lumbar puncture could be completed. In our retrospective cohort of patients with first clinical relapse indicating a demyelinating disease, methylprednisolone was administered in 61% (65/107) of patients before lumbar puncture was performed. Since the CSF concentration of KFLC was similar between untreated patients and patients under infusion of methylprednisolone and our findings indicate a significant reduction of serum KFLC concentrations, the ratio of CSF and serum KFLC (Q KFLC) might be falsely elevated. In methods to evaluate the origin of KFLC in CSF such as KFLC index or diagrams, falsely elevated Q KFLC might falsify the results and, therefore, pretend an intrathecal inflammation [18,27]. This might be of relevance in patients with borderline results with close proximity to the Q albumin-depending threshold value. However, in our retrospective analyses of MS patients, calculating the KFLC index revealed slightly higher mean values for patients who were treated with methylprednisolone as compared with untreated patients but it did not reach significant differences. In contrast to KFLC, the amount of immunoglobulins IgG, IgM, and IgA did not change in serum after treatment with high-dose methylprednisolone in our patients. Furthermore, it was shown previously that steroids do not influence oligoclonal bands by using isoelectric focusing [28,29,30,31]. However, it was reported that all CSF immunoglobulin levels decreased significantly after methylprednisolone treatment except for the IgM [32]. On the other hand, the observed reduction of serum KFLC might be due to the following effects of methylprednisolone on lymphocytes: reduction of circulating lymphocytes by induction of apoptosis or by sequestration in the lymphoid organs, decreased T-cell stimulation of B-cells and plasma cells, and ultimately lessened response of B-cells to specific mitogens [33,34,35]. It might be speculated that these methylprednisolone effects culminate in reduced production of KFLC while the rapid renal excretion lowers the actual serum concentration.

Interestingly, we did not find significant effects of plasma exchange or immunoadsorption on serum concentrations of KFLC. Therapeutic apheresis is commonly used as second-line therapy in various neuroimmunological diseases including MS and autoimmune encephalitis after insufficient clinical response to methylprednisolone. The effects of apheresis on serum immunoglobulin concentrations have been described before and are in line with our observations of a significant decrease [36].

Since CSF samples were understandably not obtained in patients who underwent therapeutic apheresis, the effect on intrathecal synthesis can only be speculated. Spurious quantitative intrathecal immunoglobulin syntheses have been described after plasma exchange or immunoadsorption in one previous study [36]. The missing significant reduction of KFLC in serum after plasma exchange therapy is conclusive with findings of several other studies [37,38,39]. The authors stated that proteins with the molecular weight of KFLC are not effectively eliminated by plasma exchange therapy as only 16% of the total quantity is extracted [39]. Further, not only the short half-life period indicating a rapid re-synthetization of KFLC in serum but also the re-equilibration of extravascular and intravascular KFLC might be reasons for the missing reduction of KFLC [39,40]. Since there are studies that reveal a significant reduction of KFLC in serum by plasma exchange, the questions whether and how a significant reduction of KFLC by plasma exchange occurs is still not entirely to answer [41,42,43].

On the other hand, the missing decrease of serum KFLC using immunoadsorption is expectable as during an immunoadsorption therapy the high affinity of staphylococcal-protein-A-columns to the Fc portion of IgG is used to selectively remove IgG antibodies, aggregated IgG, and IgG-containing immune complexes [44]. It, therefore, suggests itself that the functional principle of immunoadsorption does not allow a significant removal of proteins without an Fc portion, like KFLC.

Intravenous immunoglobulins are commonly used in a wide spectrum of autoimmune diseases including immune-mediated neuropathies and anti-*N*-methyl-D-aspartate (NMDA) receptor encephalitis [45]. Intravenous immunoglobulins are a pool of highly purified polyvalent antibodies consisting of greater than 95% IgG [46]. It is self-explanatory that treatment with intravenous immunoglobulins leads to an increase of immunoglobulin concentrations in serum, foremost IgG but also IgM. In addition to clearly higher IgG levels, KFLC concentrations in serum were slightly higher after treatment, but it did not reach a significant difference. We found a significant decrease in serum albumin after treatment with immunoglobulins, which is in line with previous studies [47]. Although the reason for the reduction in albumin remains unknown, some authors speculated that an increase in immunoglobulin concentration consequently increases systemic protein levels and creates an oncotic pressure imbalance with peripheral edema, while others suggest inhibition of the FcRn, which binds to albumin and thus extends its lifespan [47].

Serum-free light chains concentrations are supposed to have a short half-life of 2–6 h in vivo, which is in contrast to immunoglobulins having a half-time of 21 days [48]. However, our results indicate stability of KFLC in vitro for at least two weeks at 4 °C and at room temperature. Furthermore, measurements of serum and EDTA samples from the same patients did not differ in KFLC concentrations. Traumatic lumbar puncture with blood contamination of CSF samples is another common problem in clinical practice. Previous studies have shown that CSF protein analytics are only reliable up to a certain limit of blood contamination [49]. We found that addition of blood to CSF (containing up to 20,000 erythrocytes per mL CSF) did not change KFLC concentrations in CSF.

One limitation of this study is the retrospective approach when analyzing CSF and serum samples of patients who presented for the first time with symptoms suggestive for a MS relapse. The limited number of patients who were retrospectively and prospectively analyzed is caused by the strict inclusion criteria, which also hazards a selection bias. Therefore, the results should be confirmed in further studies with larger cohorts of patients.

In conclusion, our results show prominent effects of steroid therapy on KFLC concentrations, which should be considered while interpreting results. In contrast, various other pre-analytical conditions including therapeutic apheresis did not influence KFLC concentrations indicating stability of this biomarker.

## Figures and Tables

**Table 1:** Demographic data of prospective patients: treatment and different pre-analytical conditions. Percentage of females and age of patients treated with intravenous methylprednisolone, plasma exchange, immunoadsorption, and intravenous immunoglobulins were recorded. Percentage of females and age of patients whose samples were used to investigate pre-analytical conditions as storage of blood in EDTA and serum tubes, storage of CSF and serum samples for 14 days at room temperature or at 4 °C, and contamination of cerebrospinal fluid (CSF) with 5000 and 20,000 erythrocytes/mL CSF. Patient age was depicted as median and range.

Treatment or Pre-Analytic Condition	Females, n (%)	Median Age, Years (Range)
Intravenous methylprednisolone	10/16 (63%)	41.5 (18–70)
Plasma exchange	5/12 (42%)	50.5 (24–80)
Immunoadsorption	6/10 (60%)	31 (19–52)
Intravenous immunoglobulin	4/10 (40%)	63 (45–76)
Sample method (EDTA or serum tube)	17/33 (52%)	45 (25–84)
Storage time and temperature	8/16 (50%)	44.5 (25–80)
Blood contamination	11/17 (65%)	44 (32–84)

## Supplementary Materials

The following are available online at https://www.mdpi.com/2073-4409/9/4/842/s1. Table S1. Diagnosis, number of patients and immunosuppressive treatment prior to 4 weeks and to 5 to 12 weeks before samples were obtained in patients treated with intravenous methylprednisolone, plasma exchange, immunoadsorption, and intravenous immunoglobulins. Diagnosis, number of patients and immunomodulatory treatment prior to 4 weeks and to 5 to 12 weeks before samples were taken to investigate sample method, storage time and temperature, and contamination of cerebrospinal fluid (CSF) with blood. * Samples originated from 38 different patients who underwent lumbar puncture for routine diagnosis. Numbers in parenthesis represent the total number of patients with the diagnosis. Actually included patients for each experiment are listed in column “Number of patients”. Surplus sample material of these patients was used for all three experimental series. ** Included patients were diagnosed with inflammatory polyneuropathies (n = 6), MS (n = 5), myelitis (n = 4) and systemic lupus erythematodes (SLE) with involvement of the central nervous system (n = 1); Figure S1. Albumin concentrations after methylprednisolone. Albumin concentrations and quotients of patients diagnosed with multiple sclerosis according to the McDonald criteria of 2017 and clinically isolated syndrome who converted to multiple sclerosis during follow-up. Each dot represents a single measurement of a patient who was untreated or received intravenous methylprednisolone with the indicated dose. Depicted are serum concentrations (A), cerebrospinal fluid (CSF) concentrations (B) and CSF-serum albumin quotient (Q albumin) (C) in untreated patients (n = 42) and patients who received methylprednisolone infusion of 1000 mg (n = 8), 2000 mg (n = 25), 3000 mg (n = 16), 4000 mg (n = 9), or 5000 mg (n = 7). p-values of significant differences are illustrated as asterisks.; Figure S2. Kappa free light chain (KFLC) index and immunoglobulin quotients after methylprednisolone. Indices and quotients of patients diagnosed with multiple sclerosis according to the McDonald criteria of 2017 and clinically isolated syndrome who converted to multiple sclerosis during follow-up. Each dot represents a single measurement of a patient who received intravenous methylprednisolone with the indicated dose. Depicted are kappa free light chain (KFLC) index (A), CSF and serum quotients of immunoglobulin type G (IgG) (B), IgA (C) and IgM (D) in untreated patients (n = 42) and patients who received methylprednisolone infusion of 1000 mg (n = 8), 2000 mg (n = 25), 3000 mg (n = 16), 4000 mg (n = 9), or 5000 mg (n = 7). p-values of significant differences are illustrated as asterisks.; Figure S3. Serum albumin concentrations in prospective patients. Serum albumin concentrations of patients whose samples and data were collected in 2018 and 2019 are shown. Administered treatment was 1000 mg of intravenous methylprednisolone per day, one cycle of plasma exchange or immunoadsorption per day and intravenous immunoglobulins (IVIG) in a daily dosage of 30–40 g. Patients treated with 3 cycles of plasma exchange therapy (n = 5) or 3 days of intravenous methylprednisolone (n = 7) are depicted as blue dots, while patients with 5 cycles of plasma exchange therapy (n = 7) or 5 days of intravenous methylprednisolone (n = 9) are depicted as red dots. Serum concentration of albumin was determined before all treatments and after 24, 48 and 96 h of treatment with intravenous methylprednisolone (A), after 24, 48, 72, 96 and 120 h of treatment with plasma exchange (B) or immunoadsorption (n = 10) (C) and after treatment with intravenous immunoglobulins in a total dosage of 60–160 g (n = 10) (D). p-values of significant differences are illustrated as asterisks.; Figure S4. Kappa free light chain (KFLC) concentrations under different pre-analytical conditions. Concentration of KFLC of patients whose samples and data were collected in 2018 and 2019 are shown. Depicted are KFLC concentrations of blood samples stored in EDTA and serum tubes (n = 33) (A), serum (B) and cerebrospinal fluid (CSF) (C) concentrations of KFLC after storage for 14 days at room temperature or at 4°C (n = 16), and concentrations of KFLC in cerebrospinal fluid of samples contaminated with blood including 5000 and 20000 erythrocytes / ml CSF (n = 17) (D). *p*-values of significant differences are illustrated as asterisks.

## Abbreviations

KFLC	Kappa free light chains
CSF	cerebrospinal fluid
MS	multiple sclerosis
CIS	clinically isolated syndrome
Ig	immunoglobulin
IVIG	intravenous immunoglobulin
NMDA	*N*-methyl-D-aspartate
