# Supplementary figures and images for: The Impact of Immunomodulatory Treatment on Kappa Free Light Chains as Biomarker in Neuroinflammation
Source: Cells. 2020 Mar 31;9(4):842. doi: 10.3390/cells9040842 (PMC7226742; doi:10.3390/cells9040842)

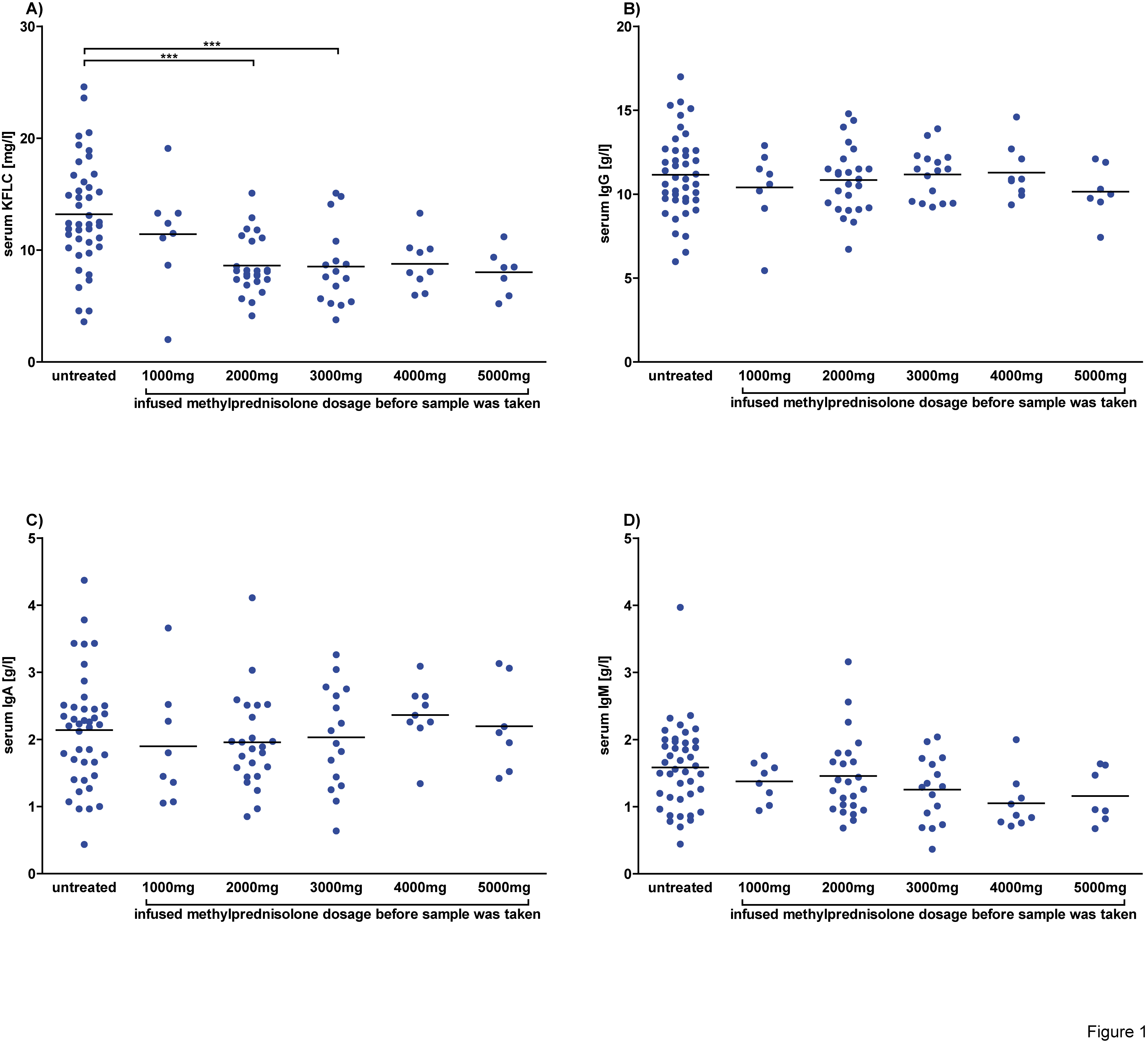

Supplement: Supplementary file 1 [file cells-09-00842-s001.zip › Figure 1.tif]

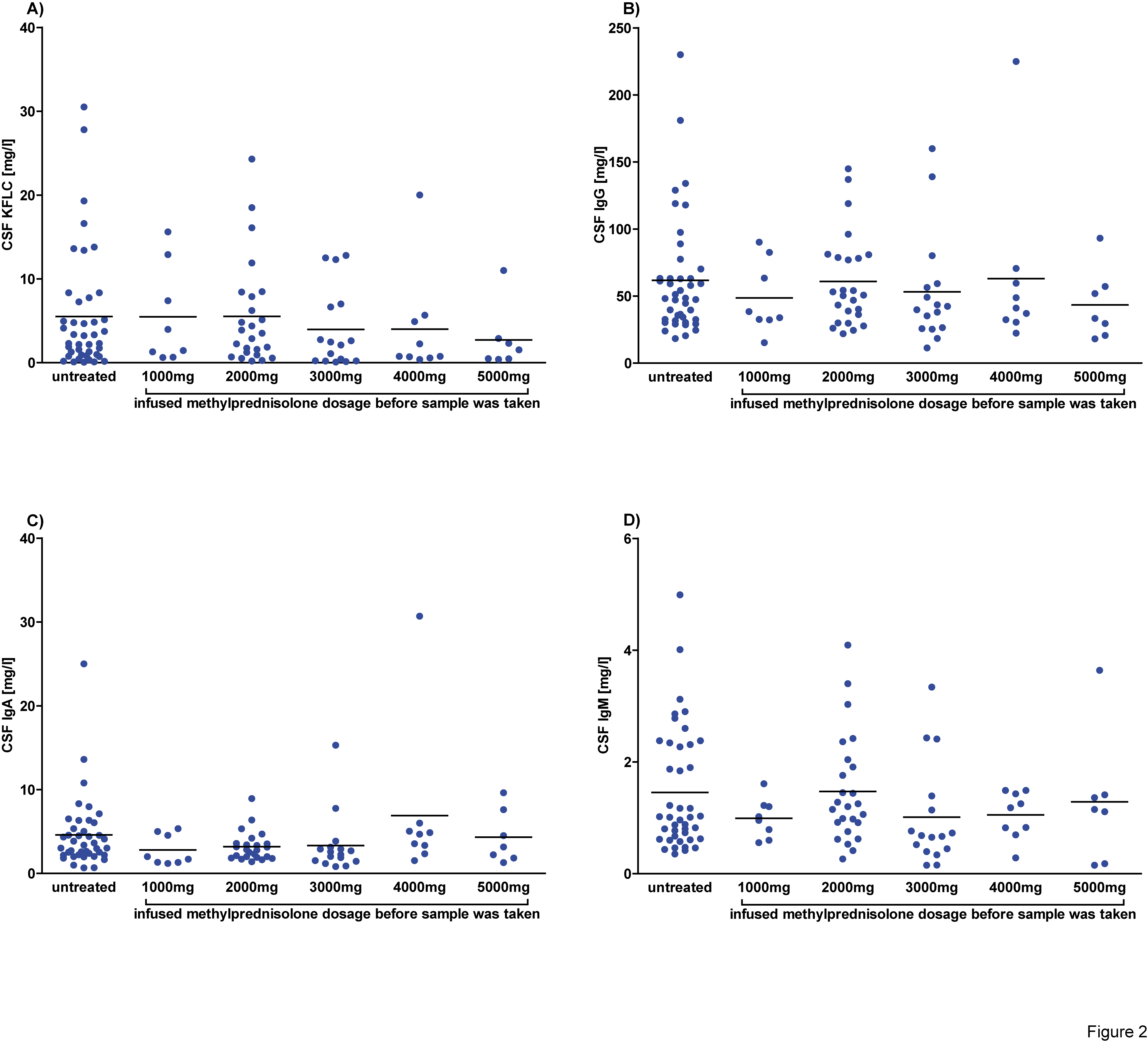

Supplement: Supplementary file 1 [file cells-09-00842-s001.zip › Figure 2.tif]

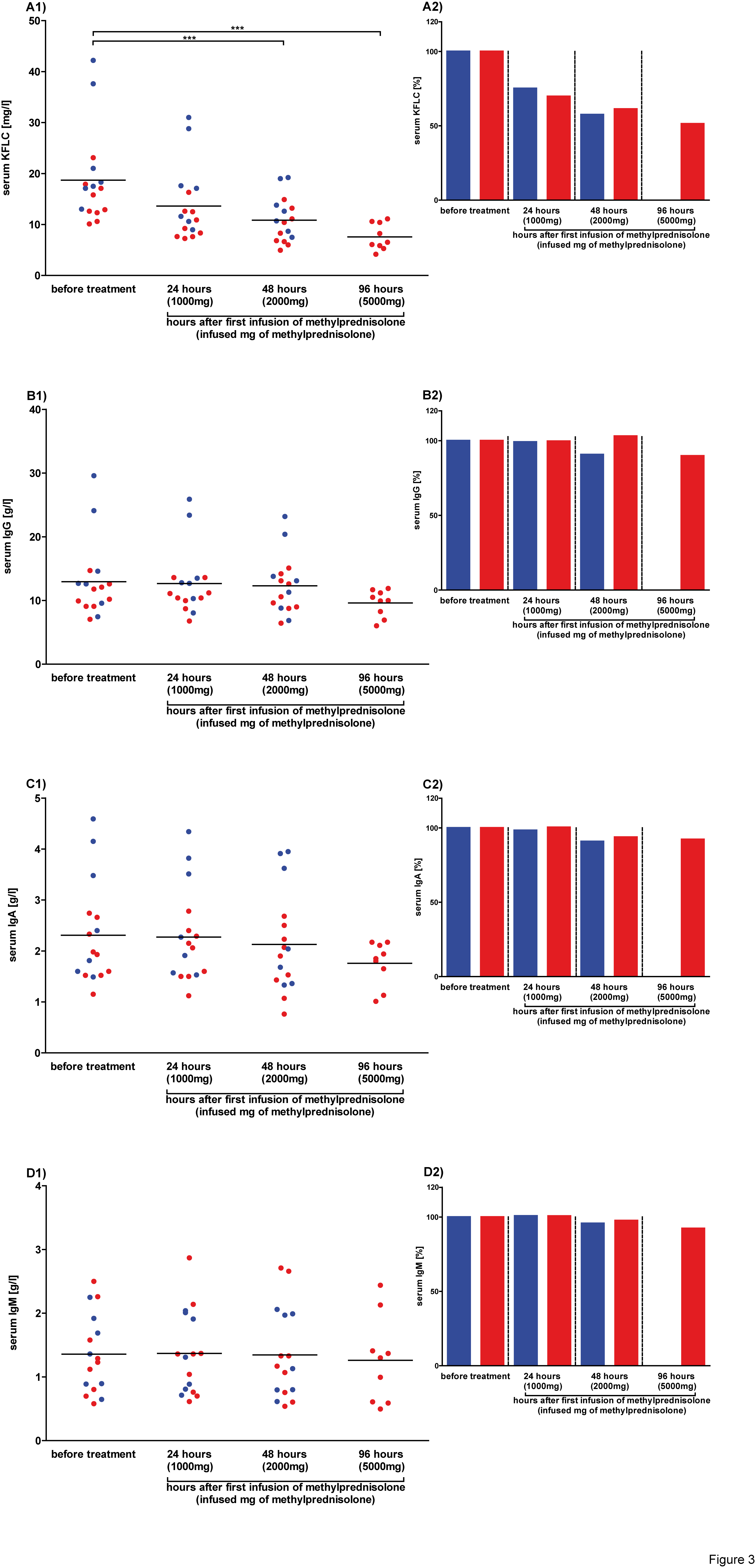

Supplement: Supplementary file 1 [file cells-09-00842-s001.zip › Figure 3.tif]

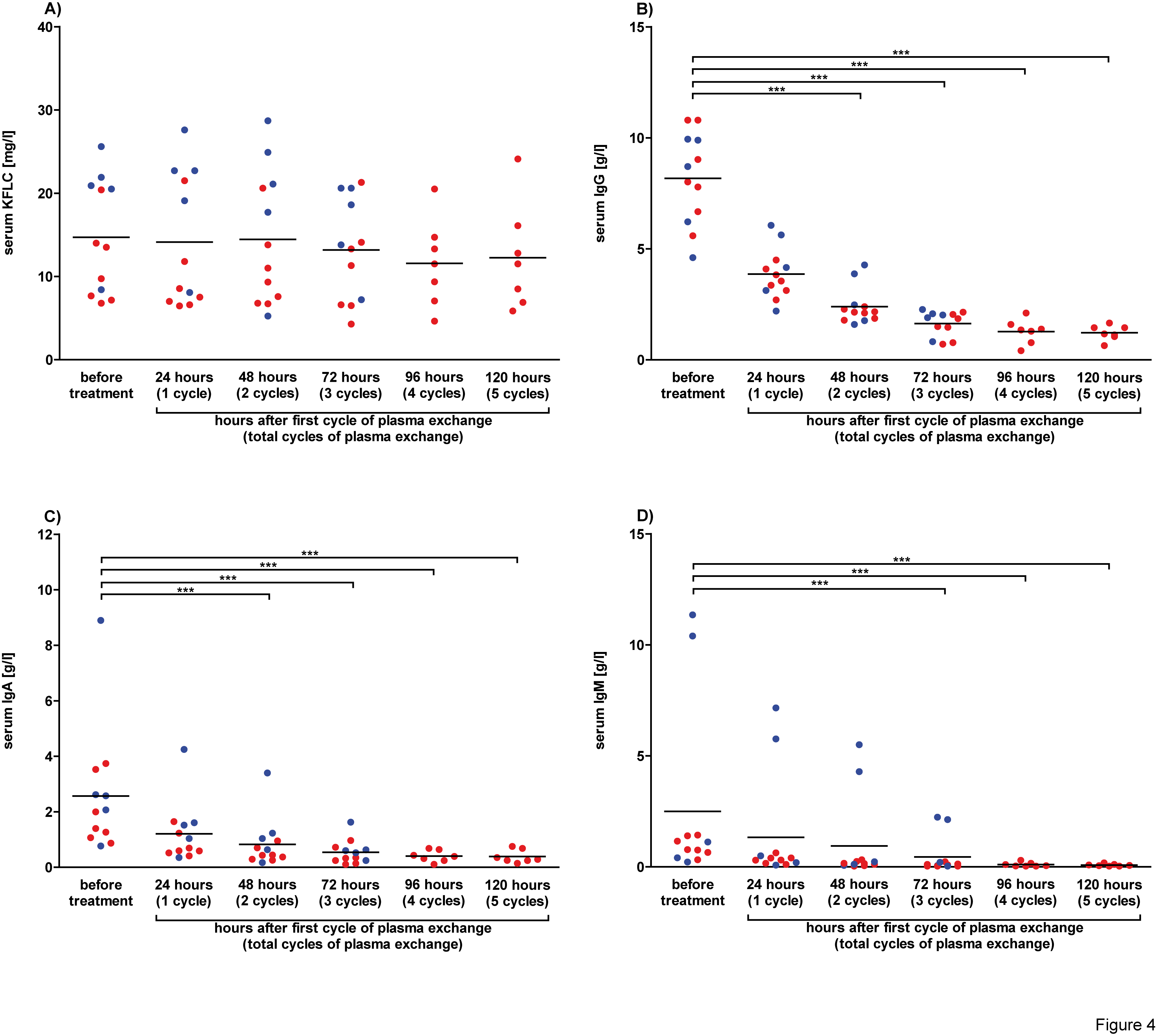

Supplement: Supplementary file 1 [file cells-09-00842-s001.zip › Figure 4.tif]

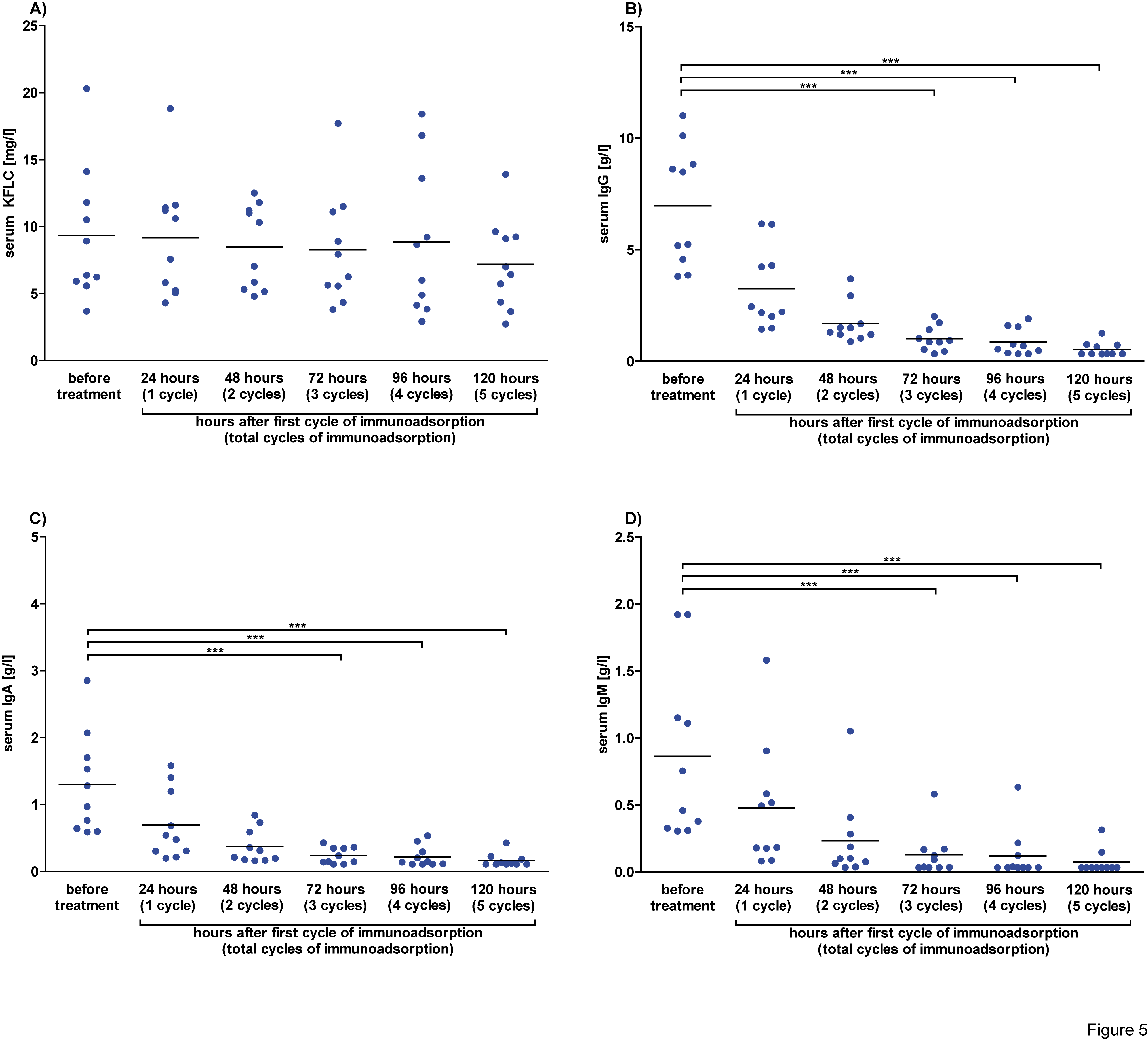

Supplement: Supplementary file 1 [file cells-09-00842-s001.zip › Figure 5.tif]

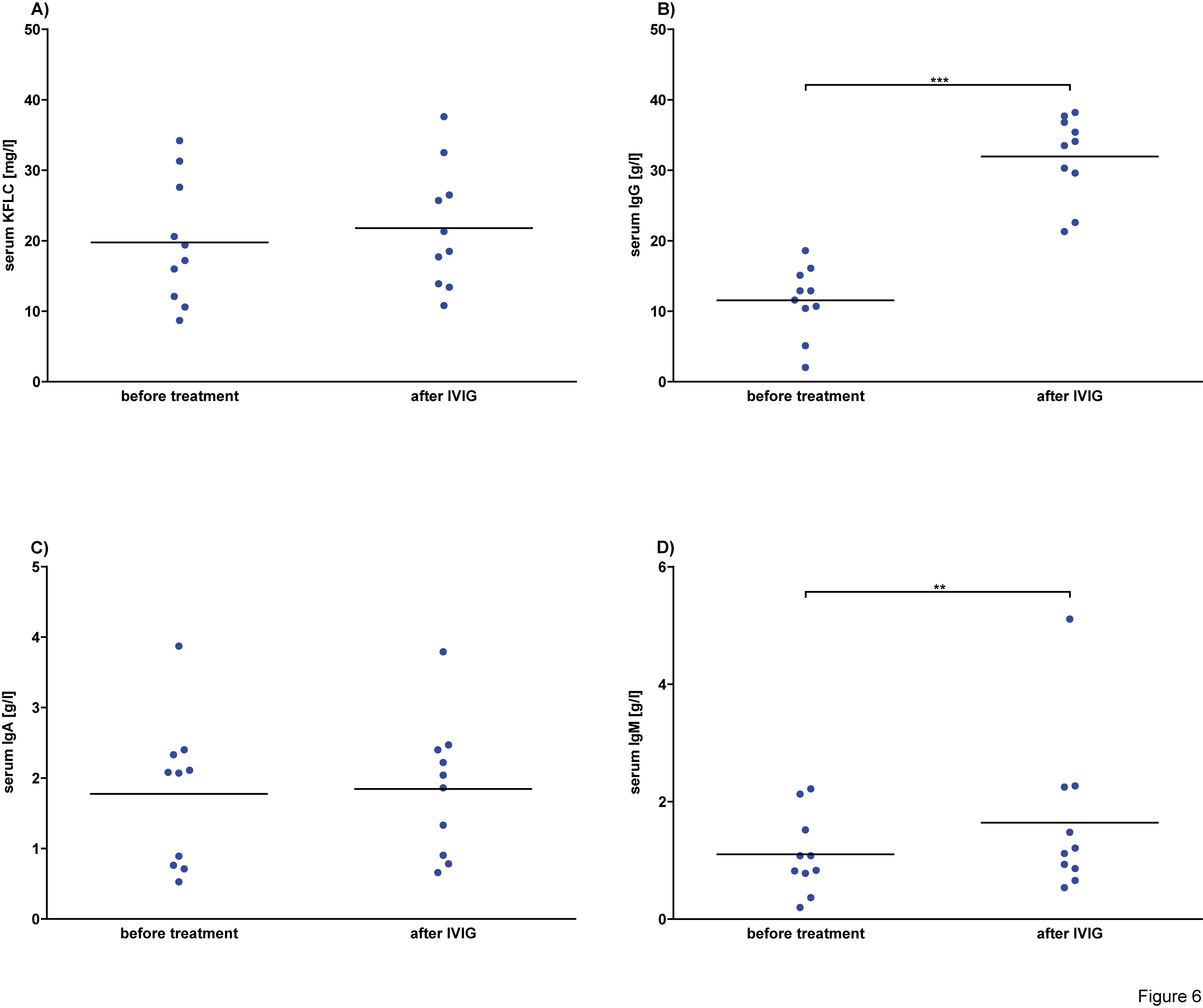

Supplement: Supplementary file 1 [file cells-09-00842-s001.zip › Figure 6.tif]

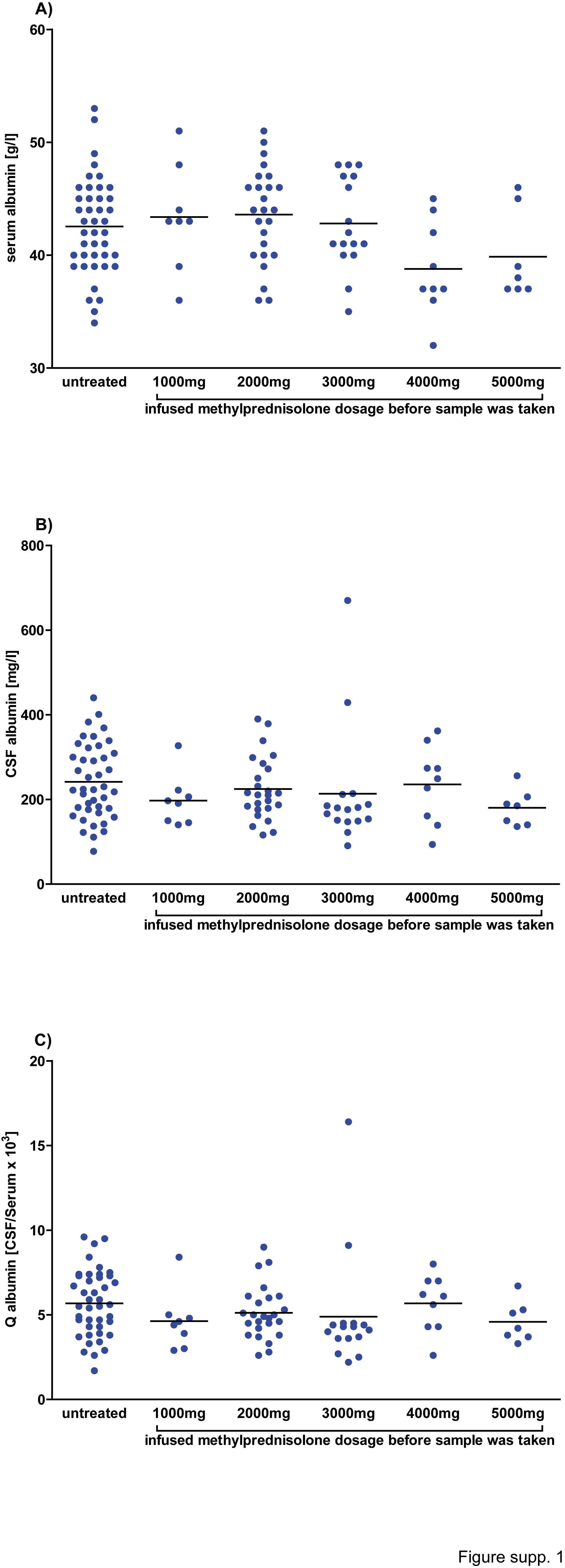

Supplement: Supplementary file 1 [file cells-09-00842-s001.zip › Figure supp. 1.tif]

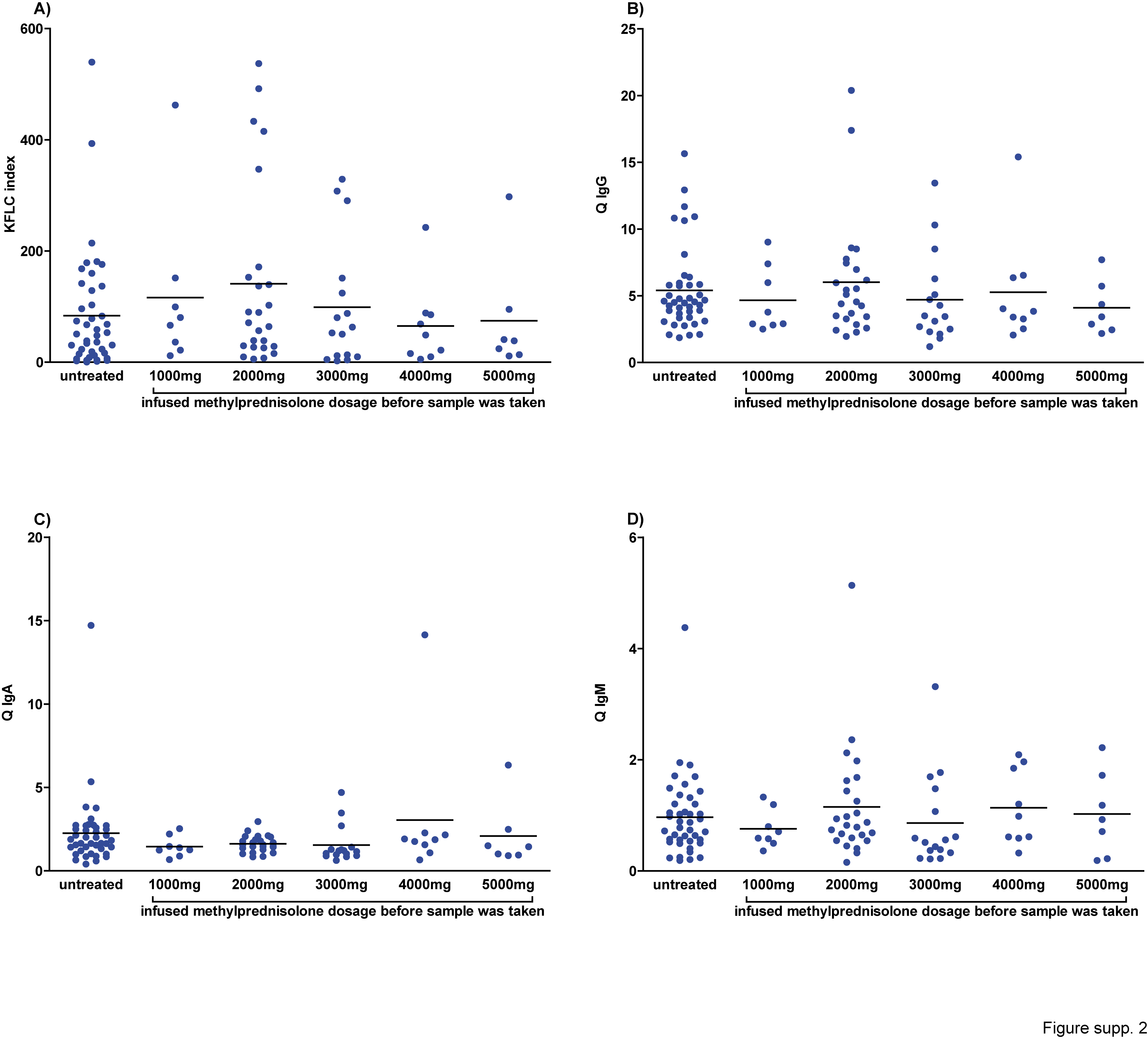

Supplement: Supplementary file 1 [file cells-09-00842-s001.zip › Figure supp. 2.tif]

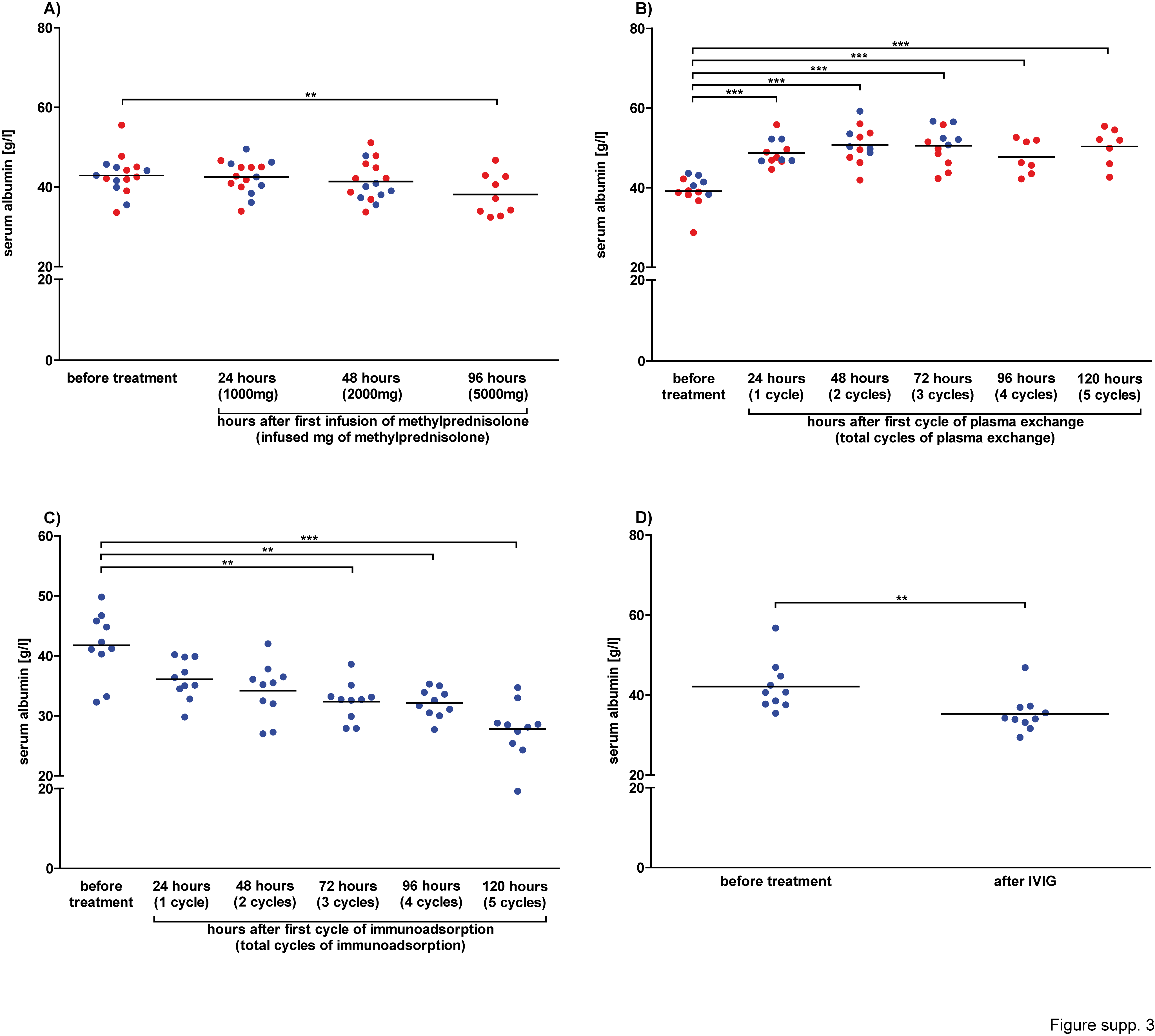

Supplement: Supplementary file 1 [file cells-09-00842-s001.zip › Figure supp. 3.tif]

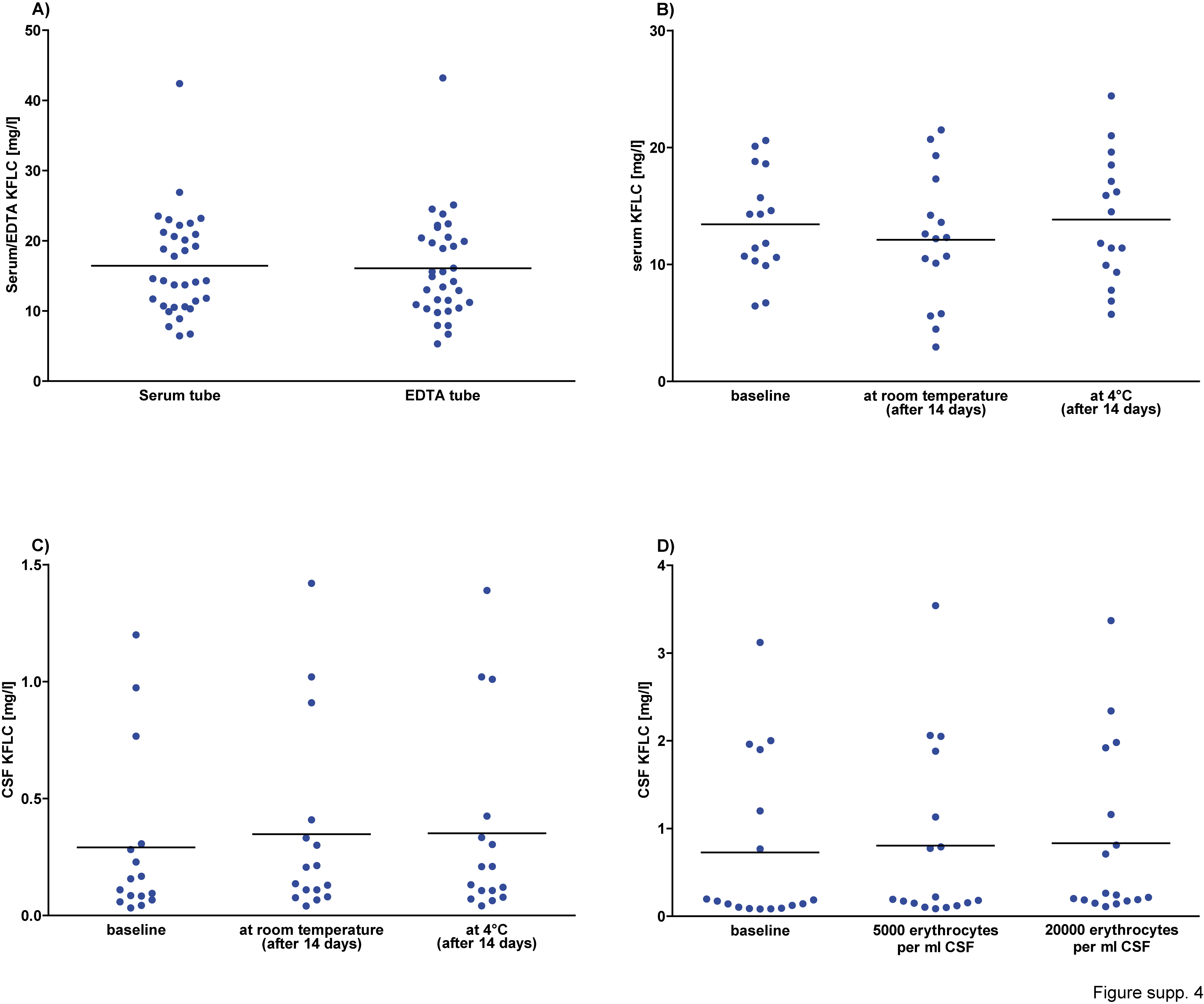

Supplement: Supplementary file 1 [file cells-09-00842-s001.zip › Figure supp. 4.tif]

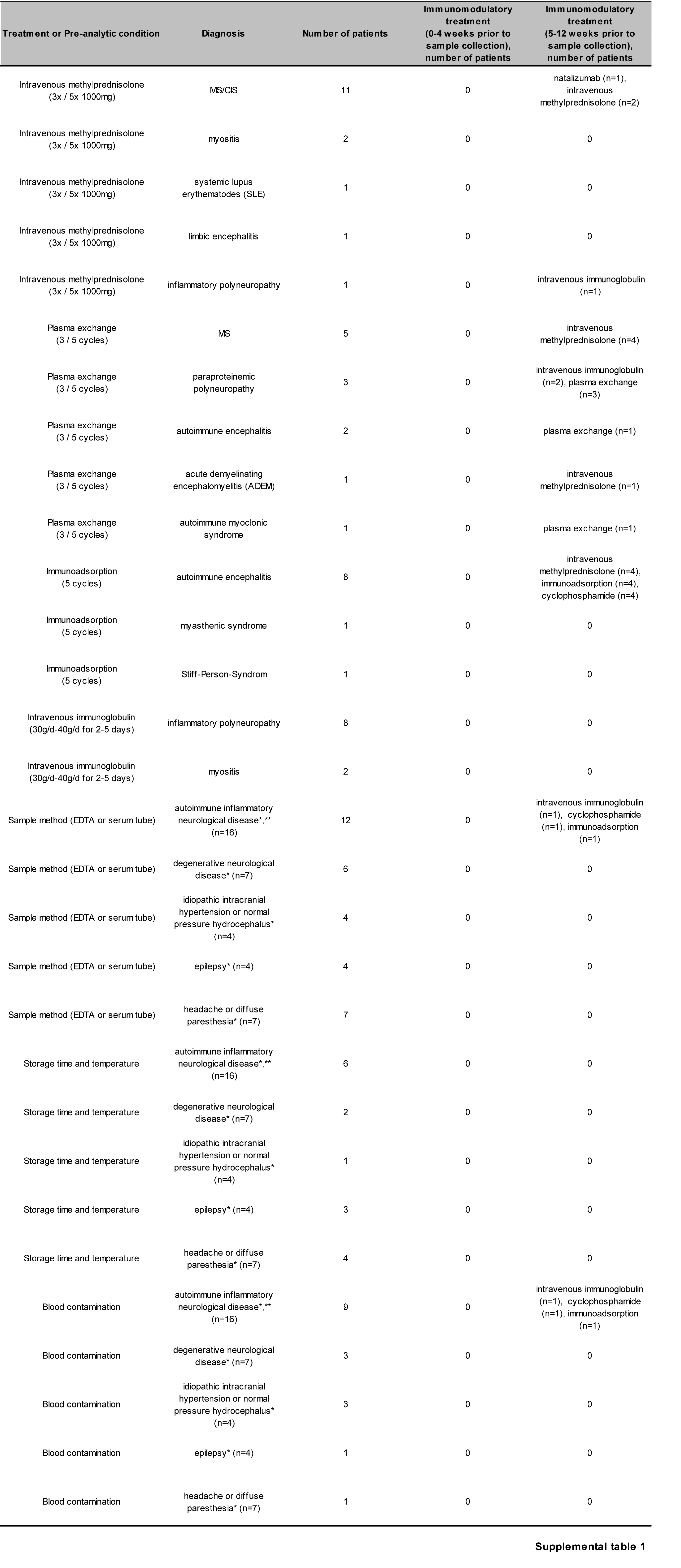

Supplement: Supplementary file 1 [file cells-09-00842-s001.zip › Supplemental Table 1.tif]

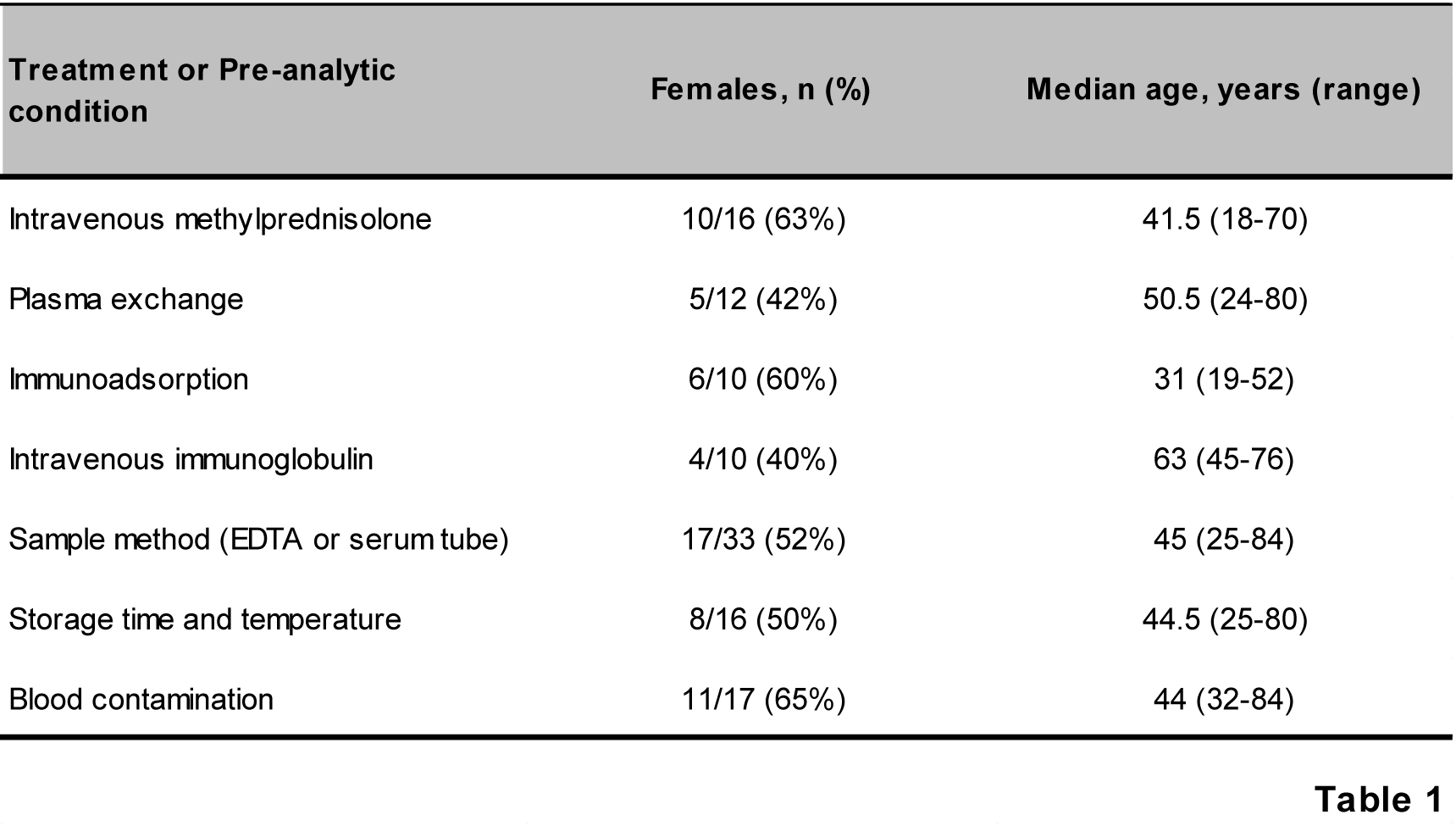

Supplement: Supplementary file 1 [file cells-09-00842-s001.zip › Table 1.tif]
